# Reduced colony‐stimulating factor 1 receptor expression in myeloid cells has limited impact on chronic lymphocytic leukaemia progression

**DOI:** 10.1111/bjh.70700

**Published:** 2026-07-26

**Authors:** Natascha Rosen, Guillermo Rodriguez‐Real, Alexander F. vom Stein, Luca D. Schreurs, Trong‐Hieu Nguyen, Viktoria Kohlhas, Thanh‐Tung Truong, Maximilian Koch, Sebastian Reinartz, Sumiya Iqbal, Shaista Ilyas, Sanjay Mathur, Nina Reinart, Phuong‐Hien Nguyen, Michael Hallek

**Affiliations:** ^1^ Department I of Internal Medicine, Faculty of Medicine and University Hospital Cologne, Center for Integrated Oncology Aachen Bonn Cologne Duesseldorf, Center for Molecular Medicine Cologne, CECAD Center of Excellence on Cellular Stress Responses in Aging‐Associated Diseases University of Cologne Cologne Germany; ^2^ Institute of Molecular Medicine RWTH Aachen University Aachen Germany; ^3^ Institute of Inorganic and Materials Chemistry University of Cologne Cologne Germany; ^4^ Center for Molecular Medicine Cologne Cologne Germany

**Keywords:** chronic lymphocytic leukaemia, CSF1R, macrophages, monocytes

## Abstract

Targeting the colony‐stimulating factor 1 receptor (CSF1R) to remove tumour‐associated macrophages is being explored as cancer therapy. This strategy may be relevant for chronic lymphocytic leukaemia (CLL), which strongly depends on support from myeloid cells. However, it is unclear how CSF1R expression affects the CLL microenvironment and leukaemic progression. To examine this question, we created *Eμ‐TCL1* transgenic mice for CLL with *Csf1r* haploinsufficiency to investigate changes in myeloid cells, Csf1r expression and leukaemia progression. *Csf1r* haploinsufficiency reduced Csf1r expression on circulating monocytes, but did not significantly impair its expression in tissue macrophages or change the overall numbers of monocytes or macrophages in blood and lymphoid tissues. *Eμ‐TCL1* transgenic mice with lower Csf1r levels had less leukaemia during early disease, but this effect faded with disease progression, and their overall survival was similar to controls. Furthermore, in vitro co‐culture demonstrates that depletion of CSF1R expression on cell lines did not affect the survival or migration of patient‐derived CLL cells. In summary, our results show that while the CSF1R pathway is important for maintaining the myeloid cells that support CLL, simply reducing CSF1R expression has only a limited effect on disease progression. Attempts to target the CSF1R for leukaemic therapy might benefit from a stronger depletion of macrophages or the combination with other agents.

## INTRODUCTION

Chronic lymphocytic leukaemia (CLL) is the most common leukaemia in the Western world. It is characterized by the monoclonal expansion and accumulation of mature, yet immunodeficient, cluster of differentiation 5^+^ B cells in the blood, lymphoid tissues and bone marrow.^1^

Although CLL cells persist long‐term in patients, they rapidly undergo apoptosis in vitro.^2^ This is explained by the importance of the tumour microenvironment (TME), which provides essential survival signals.^3^ Among TME components, macrophages play a central role in CLL progression and treatment responses.^4^ Increased monocyte counts in blood correlate with poor prognosis.^5^, ^6^ Peripheral monocytes, upon contact with leukaemic cells, differentiate into nurse‐like cells (NLCs) that prevent apoptosis and support CLL cell viability.^7^ These supportive features can be induced by leukaemia‐derived factors such as HMGB1 and NAMPT.^8^, ^9^ In return, leukaemia‐associated macrophages contribute to CLL cell survival and disease progression via secretion of soluble factors such as CD14 and macrophage migration inhibitory factor (MIF), inducing kinase signalling such as Janus kinase / Signal Transducer and Activator of Transcription (JAK/STAT) and mitogen‐activated protein kinase (MAPK) and enhancing leukaemic NF‐kB signalling,^5^, ^10^, ^11^, ^12^, ^13^, ^14^ making them attractive therapeutic targets.

A central regulator of TME organization is colony‐stimulating factor 1 (CSF‐1), a cytokine elevated in CLL patients.^15^ CSF‐1 is critical for the survival, proliferation and differentiation of mononuclear phagocytes,^16^ acting exclusively through the CSF1 receptor (CSF1R).^17^ CSF1R is expressed on monocytes, macrophages and several other cell types, including osteoclasts, neurons and cells of the female reproductive tract.^18^ In mice, complete Csf1r deficiency leads to substantial loss of macrophages, skeletal abnormalities including toothlessness, growth retardation and reduced survival, with strain‐dependent variability.^17^, ^19^

In normal B‐cell development, CSF1R is only expressed in early precursors^20^ and is repressed during maturation.^21^ In CLL, CSF1R is not aberrantly expressed on leukaemia cells,^22^ and its levels appear similar in monocytes and NLCs.^23^ However, pre‐clinical studies have shown that blocking CSF1R can disrupt macrophage support and trigger apoptosis in CLL cells. For example, treatment with anti‐Csf1r antibody in Rag2^−/−^ γc^−/−^ mice transplanted with MEC‐1 cells reduced macrophage numbers, decreased tumour burden and caused delocalization of CLL cells from tissues to blood, enhancing their sensitivity to anti‐CD20 therapy.^24^ Similar effects were observed in vitro using small‐molecule inhibitors (GW‐2580, ARRY‐382) or antibodies targeting CSF1R.^23^, ^24^ These studies primarily aimed to deplete macrophages but did not investigate whether modulating CSF1R expression levels could influence disease development.

To address this gap, we investigated the role of CSF1R expression level in CLL by adopting a genetic approach: we used heterozygous *Csf1r* ^+/−^ mice, which are viable despite reduced Csf1r expression, thus avoiding the embryonic lethality observed in Csf1r^−/−^ animals.^17^ *Csf1r* ^+/−^ mice were crossed with the *Eμ‐TCL1* transgenic mice, serving as a model for CLL,^25^, ^26^ to examine the effects of different Csf1r expression levels in a physiologically relevant setting.

## MATERIALS AND METHODS

### CLL samples

Primary CLL samples were collected from peripheral blood with informed consent, following the Declaration of Helsinki and approved by the Institutional Ethics Committee (no. 11‐319 and no. 19‐1438_1) of the University Hospital Cologne. Peripheral blood mononuclear cells (PBMCs) and CLL cells were cryopreserved at −150°C and freshly thawed for each assay.

### Mouse experiments

*Csf1r* ^+/−^ mice^17^ on C57BL6J x C3Heb/FeJ background (kind gift from E. Richard Stanley, NYC, USA) were crossed with *Eμ‐TCL1* transgenic mice^27^ on C57BL/6J background. The resulting *TCL1* ^*tg*/*wt*^ *Csf1r* ^+/−^ and control *TCL1* ^*tg*/*wt*^ *Csf1r* ^+/+^ mice were housed in a conventional pathogen‐free facility at the University Hospital Cologne.

For survival experiments, mice were monitored from birth until death or euthanasia according to approved animal welfare guidelines. Blood was collected at months 2, 4, 6, 8, 10 and 12 via tail vein incision into ethylenediaminetetraacetic acid (EDTA)‐coated microvettes (Sarstedt) and kept refrigerated until analysis.^11^, ^13^, ^28^

All mouse procedures were approved by the North Rhine‐Westphalia state authorities Landesamt für Natur, Umwelt und Verbraucherschutz (LANUV: 84‐02.04.2014.A146; 81‐02.04.2019.A009).

### Cell culture

MacCsf1r^+/+^ and MacCsf1r^−/−^ murine macrophage cell lines (Yu et al., 2008) were kindly provided by E. Richard Stanley (NYC, USA) and cultured in Dulbecco's Modified Eagle's Medium (DMEM) (Gibco) containing 10% fetal bovine serum (FBS) (PAN‐Biotech), 1% Pen‐Strep (Gibco) and mouse granulocyte‐macrophage colony‐stimulating factor (GM‐CSF) (BioLegend) at concentrations indicated for each experiment below.

THP‐1 monocyte cell line (RRID:CVCL_0006) was purchased from German Collection of Microorganisms and Cell Cultures GmbH (DSMZ) (ACC16) and cultured in Roswell Park Memorial Institute medium (RPMI) (Gibco) supplemented with 10% FBS and 1% Pen‐Strep.

### Generation of *CSF1R* knockout THP‐1 cells via Clustered Regularly Interspaced Short Palindromic Repeats/CRISPR‐associated protein 9 (CRISPR/Cas 9)

The THP‐1 *CSF1R* ^−/−^ cells were generated in two steps. First, THP‐1 cells (1 × 10^6^) were infected twice with third‐generation lentiviral vectors encoding Cas9‐eGFP (Thermo Fisher) at multiplicity of infection (MOI) 5 by spin infection (800 rpm, 120 min, 37°C), and single‐cell clones with high eGFP expression were isolated by fluorescence‐activated cell sorting (FACS).

For *CSF1R* knockout, cells were transduced with lentiviral particles containing four guide ribonucleic acid (gRNAs) targeting human *CSF1R* (Thermo Fisher; gRNA‐1: CAACGCTACCTTCCAAAACA; gRNA‐2: GAACGTGCTAGCACAGGAGG; gRNA‐3: AACGGTGACCTTGCGATGTG; gRNA‐4: GGACACTGGGCTCTATCACT). Scramble controls were generated using a non‐targeting gRNA (scr‐gRNA: GTACGTCGGTATAACTCCTC; Invitrogen LentiArray™).

Cas9‐expressing THP‐1 cells (1 × 10^4^ per well) were seeded into 96‐well plates and transduced at MOI 10 using spin infection (800 rpm, 90 min, 37°C). After 72 h, cells were washed and selected in RPMI with 0.75 μg/mL puromycin (Carl Roth) for over 7 days, replenishing medium after 3 days.

### Synthesis of doxorubicin‐loaded liposomes (Lipo‐Doxo)

Doxorubicin‐loaded liposomes were prepared using the thin‐film hydration method with 1,2‐dipalmitoyl‐sn‐glycero‐3‐phosphocholine, cholesterol and 1,2‐Distearoyl‐sn‐glycero‐3‐phosphoethanolamine‐N‐[amino(polyethylene glycol)‐2000] (DSPE‐PEG(2000)‐NH_2_) dissolved in chloroform. After solvent evaporation under reduced pressure and vacuum drying (2 h), the lipid film was hydrated with 250 mM ammonium hydrogen phosphate ((NH_4_)_2_HPO_4_) buffer at 60°C, followed by five freeze–thaw cycles and extrusion through 200 nm polycarbonate membranes to produce unilamellar vesicles.

The external buffer was exchanged for phosphate‐buffered saline (PBS) (pH 7.4) via Sephadex G‐50 size exclusion chromatography (SEC) to establish a pH gradient. Doxorubicin hydrochloride was added at a 1:10 drug‐to‐lipid molar ratio and incubated at 60°C for 1 h for active loading. The unencapsulated drug was removed by a second SEC step. The final formulation had a phospholipid concentration of 570.70 μg/mL (Stewart assay) and a doxorubicin loading capacity of 37.19% (high‐performance liquid chromatography [HPLC]). The two SEC steps ensured purity but contributed to partial lipid dilution.

### CLL co‐culture and viability assays

For MacCsf1r co‐cultures, 1 × 10^5^ cells were seeded in 24‐well plates and incubated overnight before adding 5 × 10^5^ cryopreserved CLL cells. Culture medium was supplemented with 200 ng/mL GM‐CSF.

In THP‐1 co‐cultures, 1 × 10^5^ THP‐1 cells were differentiated with 100 ng/mL phorbol 12‐myristate 13‐acetate (PMA; Sigma‐Aldrich) for 48 h, washed to remove PMA, and co‐cultured with 5 × 10^5^ patient‐derived CLL cells.

For Lipo‐Doxo toxicity assays, 3 × 10^5^ PBMCs from three healthy donors were seeded in 96‐well plates and treated with 0.1 μM or 1 μM Lipo‐Doxo. After 24 h, viability of total cells and immune subsets was analysed by flow cytometry.

In mixed PBMC–CLL cultures, 5 × 10^6^ PBMCs (±1 μM Lipo‐Doxo, 24 h) were co‐cultured with 5 × 10^5^ CLL cells from two patients in 24‐well plates. PBMCs were washed and resuspended in RPMI before being added to CLL cultures. Monocultures of CLL cells served as controls. At 24, 48 and 72 h, 200 μL of cell suspension from each well was transferred to 96 well plates for viability analysis. All conditions were in technical triplicates.

CLL viability was measured by flow cytometry using Annexin V antibody (BioLegend) on a MACSQuant X cytometer (Miltenyi Biotec), analysed with FlowJo (BD Pharmingen).

### Next‐generation messenger ribonucleic acid (mRNA) sequencing

THP‐1 scramble control and CSF1R‐knockout (KO) macrophages, as well as MacCsf1r^+/+^ and MacCsf1r^−/−^ cells, were subjected to short read 3′ prime bulk ribonucleic acid (RNA) sequencing analysis. THP‐1 cells were differentiated using 100 ng/mL PMA (Sigma‐Aldrich) for 48 h prior to RNA isolation. For all conditions, 1 × 10^6^ cells per replicate were seeded, with experiments performed in biological triplicates. All cell lines were maintained in RPMI containing 10% FBS and 1% Pen‐Strep; MacCsf1r^+/+^ and MacCsf1r^−/−^ cell media was further supplemented with 10 ng/mL murine GM‐CSF. Total RNA was isolated using the RNeasy Mini kit (Qiagen) according to the manufacturer's instructions. A paired‐end (2 × 100 bp) mRNA sequencing run with poly‐A selection was performed at the Cologne Center for Genomics (CCG). The Illumina TruSeq stranded New England Biolabs (NEB) protocol was used according to the manufacturer's instructions for sample preparation. The sequencing was run on a NovaSeq 6000 Sequencing system (Illumina). Gene alignment and expression quantification were performed by the nf‐core rnaseq pipeline. Differential gene expression analysis was conducted with DESEQ2. Pathway analysis was carried out with clusterProfiler. All figures are generated using R‐base functions and the ggplot2 package.

### Statistical analysis and data presentation

Statistical analyses were performed using GraphPad Prism 8.4.0. Unless stated otherwise, the Mann–Whitney test was used. Dot plots show medians; line graphs show mean ± Standard error of the mean (SEM). *p*‐values are indicated as: *≤0.05, **≤0.01, ***≤0.001, ****≤0.0001. Figures were prepared using GraphPad Prism and Adobe Illustrator.

## RESULTS

### Myeloid cell depletion impairs support of CLL cell survival

To assess whether circulating myeloid cells support CLL survival, we conducted co‐culture of patient‐derived CLL cells with PBMCs from healthy donors over 24, 48 and 72 h. To deplete myeloid cells, PBMCs were treated with liposomes encapsulating the chemotherapy drug doxorubicin (Lipo‐Doxo), which significantly reduced the viability of the CD14^+^ subset compared to the total PBMC population at 1 μM (Figure S1A). In co‐culture with CLL cells, PBMCs pre‐treated with 1 μM Lipo‐Doxo exhibited a significantly reduced capacity to support CLL cell survival compared to the Dimethyl sulfoxid (DMSO)‐treated controls (Figure S1B). Having confirmed the role of myeloid cells in supporting CLL cell survival, we next investigated the role of CSF1R, primarily expressed on monocytes and macrophages, in this crosstalk.

### Heterozygous deletion of *Csf1r* significantly reduces Csf1r expression on peripheral myeloid cells

**FIGURE 1:**
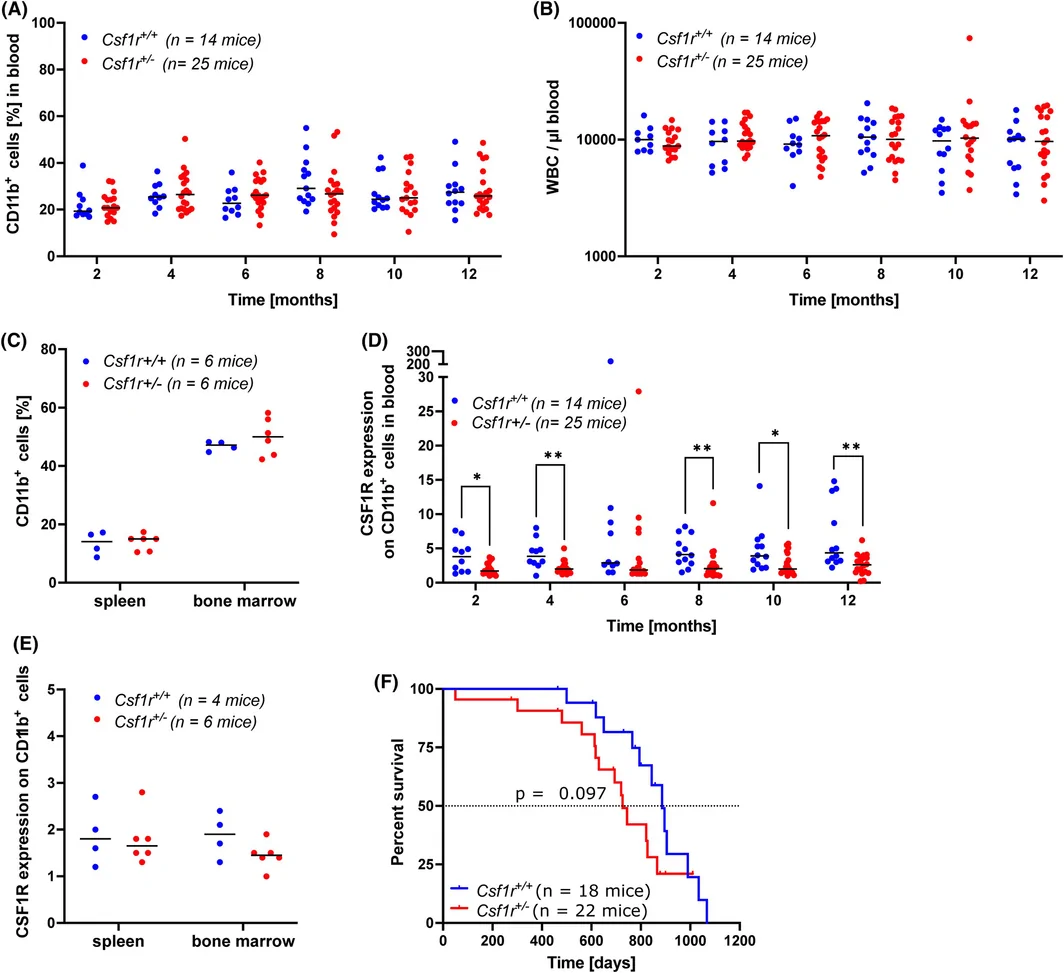
Heterozygous deletion of *Csf1r* significantly reduced Csf1r expression on peripheral myeloid cells. (A) Longitudinal analysis over 1 year showing monocyte populations (CD11b+ cells) in peripheral blood from *Csf1r* ^+/+^ and *Csf1r* ^+/−^ mice. (B) White blood cell (WBC) counts for *Csf1r* ^+/+^ and *Csf1r* ^+/−^ mice, measured using the XE‐5000 haematology analyser. (C) Flow cytometry analysis of CD11b^+^ cells in the spleen and bone marrow of 8‐ to 10‐month‐old *Csf1r* ^+/+^ and *Csf1r* ^+/−^ mice. (D) Csf1r expression on CD45^+^ CD11b^+^ peripheral blood cells over 1 year, shown as the mean fluorescence intensity (MFI) ratio normalized to the isotype control. (E) Protein expression of Csf1r on CD11b^+^ cells from the spleen and bone marrow of 8‐ to 10‐month‐old mice, expressed as the MFI ratio normalized to the FMO (fluorescence minus one). (F) Kaplan–Meier survival curves for *Csf1r* ^+/+^ and *Csf1r* ^+/−^ mice. Statistical comparison was performed using the log‐rank (Mantel–Cox) test. A cohort of *n* = 14 *Csf1r* ^+/+^ and *n* = 25 *Csf1r* ^+/−^ mice was included in the analysis of peripheral blood in panel (A, B and D). *n* = 4/*n* = 6 mice, respectively, were included for compartmental analysis in panel (C) and (E). *n* = 18/*n* = 22 mice, respectively, were included in survival analysis in panel (F).

To modulate *Csf1r* expression independently of myeloid cell depletion, we examined the myeloid compartment of *Csf1r* ^+/−^ mice, which show reduced Csf1r levels.^17^ Longitudinal blood analysis revealed comparable counts of CD11b^+^ blood monocytes and total white blood cells (WBCs) between *Csf1r* ^+/−^ and wild‐type mice (Figure 1A,B). Likewise, CD11b^+^ myeloid cell frequencies in spleen and bone marrow remained unchanged (Figure 1C).

Despite normal cell counts, *Csf1r* ^+/−^ mice displayed significantly reduced Csf1r expression on circulating CD11b^+^ monocytes (Figure 1D), while expression in spleen and bone marrow myeloid cells showed no significant differences (Figure 1E). Finally, *Csf1r* ^+/−^ mice showed a slight but non‐significant reduction in lifespan, living an average of 160 days shorter than their wild‐type littermates (Figure 1F).

**FIGURE 2:**
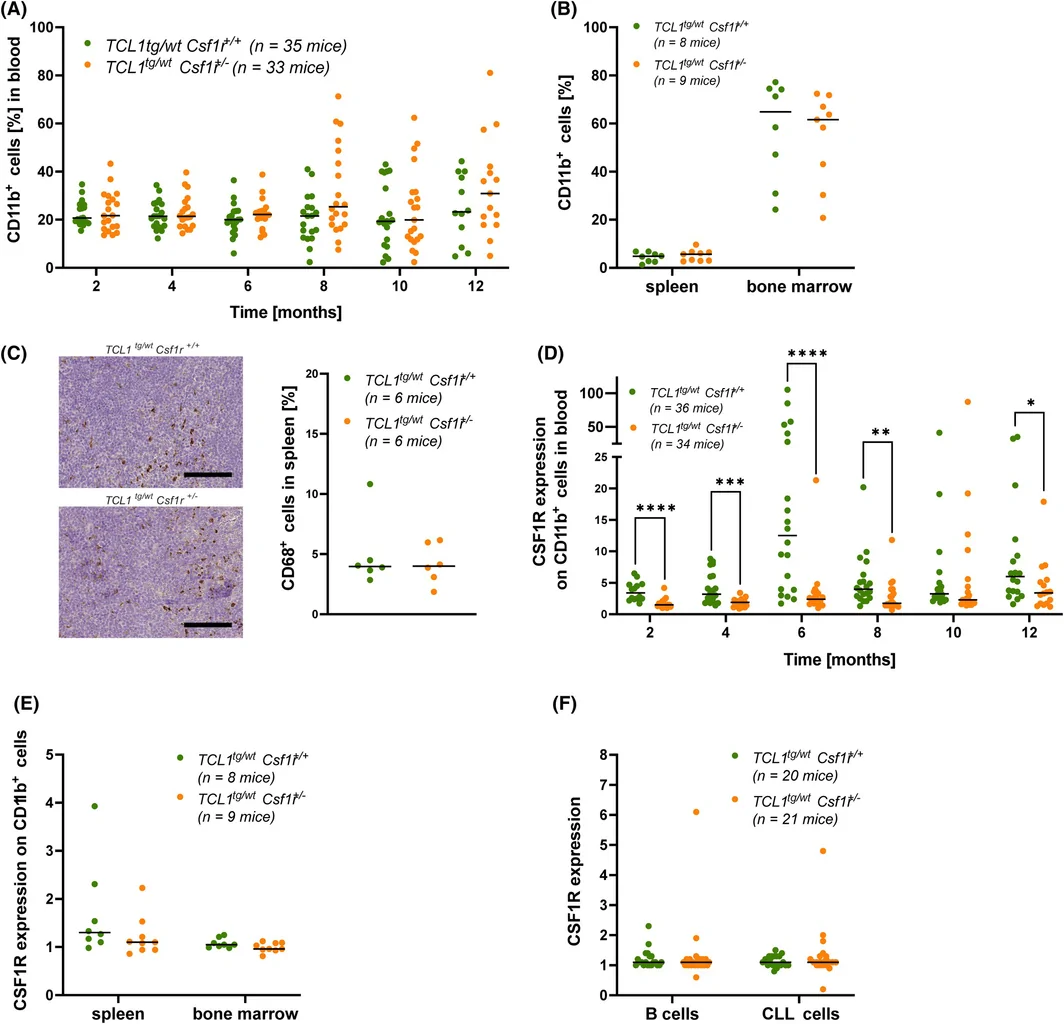
Heterozygous deletion of *Csf1r* in the *TCL1* ^*tg*/*wt*^ mice reiterated the reduced Csf1r expression on peripheral myeloid cells. (A) Longitudinal analysis of peripheral blood CD11b+ cells in *TCL1* ^*tg*/*wt*^ *Csf1r* ^+/+^ and *TCL1* ^*tg*/*wt*^ *Csf1r* ^+/−^ mice. (B) Flow cytometry analysis of CD11b^+^ cells in the spleen and bone marrow of 8‐ to 10‐month‐old *TCL1* ^*tg*/*wt*^ *Csf1r* ^+/+^ and *TCL1* ^*tg*/*wt*^ *Csf1r* ^+/−^ mice. (C) Representative immunohistochemical staining of CD68^+^ cells in spleen sections (scale bars = 100 μm) and quantification for individual mice. (D) Csf1r expression on CD11b^+^ cells in blood over 1 year, expressed as the MFI ratio normalized to the isotype control. (E) Csf1r expression on CD11b^+^ cells in the spleen and bone marrow in 8‐ to 10‐month‐old mice, expressed as the MFI ratio normalized to the FMO. (F) Flow cytometry analysis of Csf1r expression on CD45^+^ CD19^+^ CD5^−^ B cells and CD45^+^ CD19^+^ CD5^+^ CLL cells in peripheral blood, from 8‐ to 10‐month‐old mice; MFI values were calculated as in (D). A cohort of *n* = 36 *TCL1* ^*tg*/*wt*^ *Csf1r* ^+/+^ and *n* = 34 *TCL1* ^*tg*/*wt*^ *Csf1r* ^+/^ mice was included in the analysis of peripheral blood in panel (A) and (D). *n* = 8/*n* = 9 mice, respectively, were included for compartmental analysis in panel (B) and (E). Histology was performed in *n* = 6 mice each in panel C. CSF1R expression in panel (F) was measured in *n* = 20/*n* = 21 mice respectively.

To assess the impact of reduced monocytic Csf1r expression on CLL development, we crossed *Csf1r* ^+/−^ with *Eμ‐TCL1* transgenic mice to generate *TCL1* ^*tg*/*wt*^ *Csf1r* ^+/+^ and *TCL1* ^*tg*/*wt*^ *Csf1r* ^+/−^ cohorts. Consistent with *Csf1r* ^+/−^ mice, longitudinal blood analysis revealed no significant difference in monocyte counts in *TCL1* ^*tg*/*wt*^ mice regardless of their *Csf1r* genotype (Figure 2A). Similarly, myeloid cell frequencies in the spleen and bone marrow remained stable (Figure 2B), as well as CD68^+^ macrophage frequency in the spleen (Figure 2C). As observed in *Csf1r* ^+/−^ mice, Csf1r expression on blood monocytes was significantly reduced in *TCL1* ^*tg*/*wt*^ *Csf1r* ^+/−^ mice (Figure 2D), with no change in lymphoid tissue myeloid cells (Figure 2E). As expected, CLL cells^22^ and healthy B cells^20^ did not express Csf1r (Figure 2F).

### Loss of one *Csf1r* allele reduced leukaemic burden in the peripheral blood of *TCL1* ^*tg*^ ^/*wt*^ mice without affecting tissue infiltration

**FIGURE 3:**
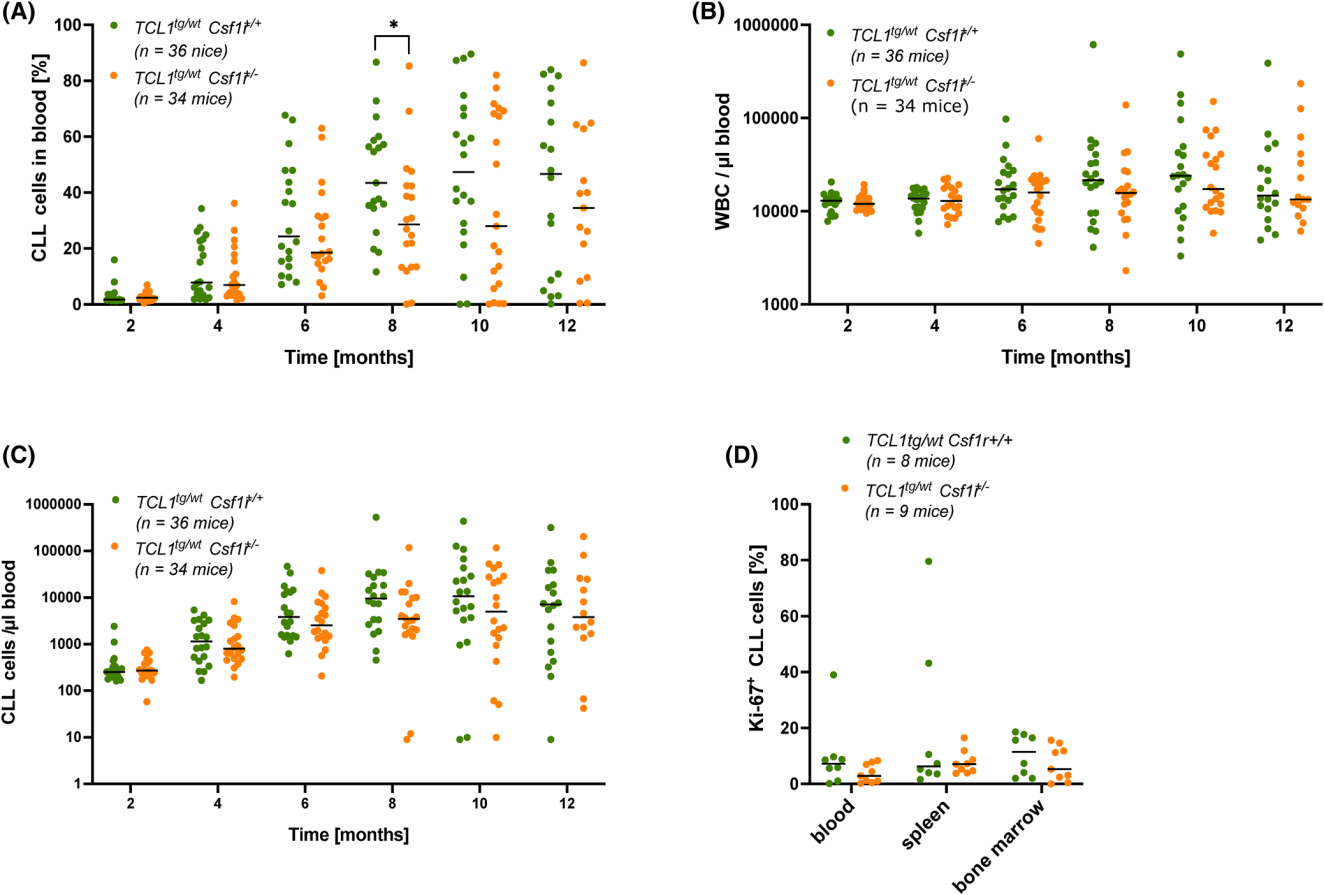
Loss of one *Csf1r* allele reduced leukaemic burden in blood but not in lymphoid tissues of *TCL1* ^*tg*/*wt*^ mice. (A) Longitudinal flow cytometric analysis of CD45^+^ CD19^+^ CD5^+^ CLL cells in the peripheral blood of *TCL1* ^*tg*/*wt*^ *Csf1r* ^+/+^ and *TCL1* ^*tg*/*wt*^ *Csf1r* ^+/−^ mice over 1 year. (B) Absolute numbers of CLL cells, calculated from the white blood cell (WBC) counts. (C) WBC counts measured with the XE‐5000 haematology analyser. (D) Proliferating CLL cells (CD19^+^ CD5^+^ Ki‐67^+^) in blood (*p* = 0.1996), spleen (*p* > 0.9999) and bone marrow (*p* = 0.2766) from 8‐ to 10‐month‐old mice. A cohort of *n* = 36 *TCL1* ^*tg*/*wt*^ *Csf1r* ^+/+^ and *n* = 34 *TCL1* ^*tg*/*wt*^ *Csf1r* ^+/^ mice was included in the analysis of peripheral blood in panel (A–C). *n* = 8/*n* = 9 mice, respectively, were included for compartmental analysis in panel (D).

Longitudinal blood analysis over 12 months showed significantly reduced CLL burden at month 8 in *TCL1* ^*tg*/*wt*^ *Csf1r* ^+/−^ mice (Mean ± SEM = 31.07 ± 4.806% CLL cells in blood) compared to *TCL1* ^*tg*/*wt*^ *Csf1r* ^+/+^ mice (Mean ± SEM = 45.74 ± 4.566% CLL cells in blood). This difference persisted, albeit less markedly, at 12 months of age (*TCL1* ^*tg*/*wt*^ *Csf1r* ^+^/^+^: 42.20 ± 7.376%; *TCL1* ^*tg*/*wt*^ *Csf1r* ^+/−^: 35.41 ± 6.679%) (Figure 3A). Although the absolute number of leukaemic cells in *TCL1* ^*tg*/*wt*^ *Csf1r* ^+/−^ mice followed the same trend, the difference did not reach statistical significance (Figure 3B), and WBC counts were comparable between genotypes (Figure 3C). Furthermore, Ki‐67 staining showed no statistically significant differences in CLL proliferation across blood, spleen and bone marrow samples between *TCL1* ^*tg*/*wt*^ *Csf1r* ^+/+^ and *TCL1* ^*tg*/*wt*^ *Csf1r* ^+/−^ mice (Figure 3D), suggesting minimal influence of Csf1r expression on CLL proliferation.

**FIGURE 4:**
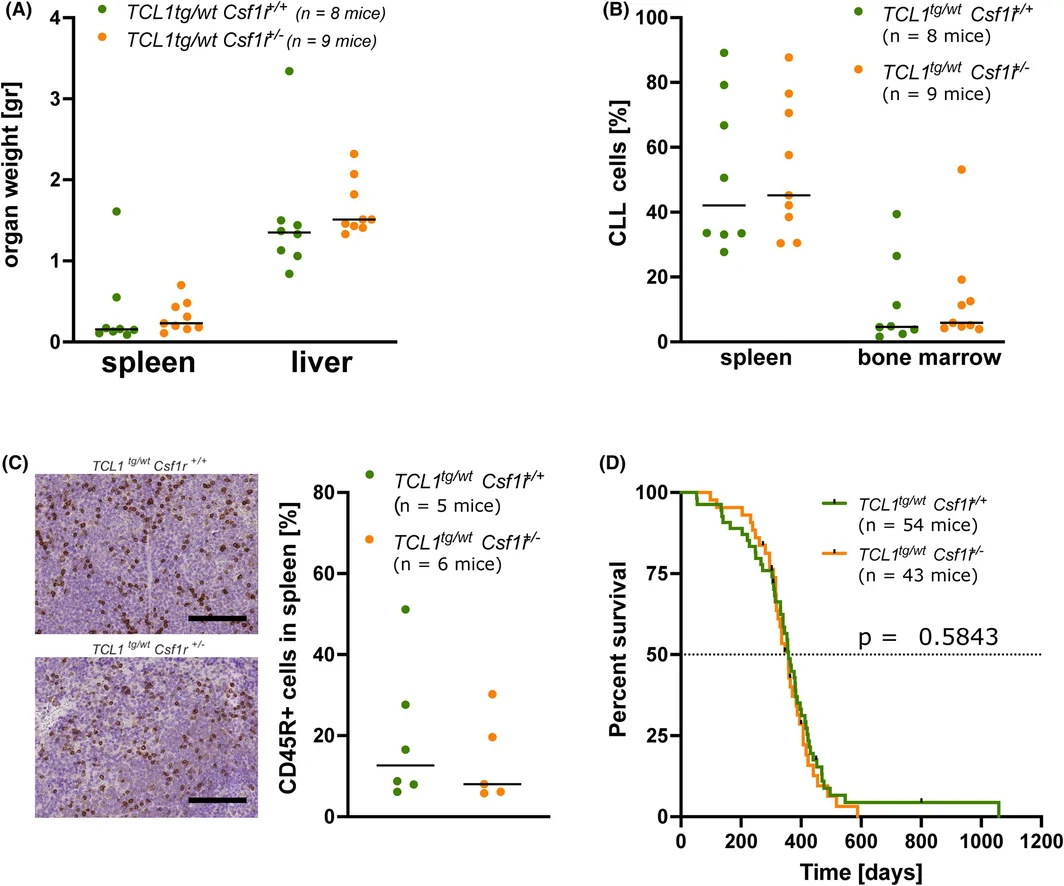
CLL development in lymphoid tissue was not affected by heterozygous *Csf1r* deletion. (A) Spleen and liver weights in 8‐ to 10‐month‐old *TCL1* ^*tg*/*wt*^ *Csf1r* ^+/+^ and *TCL1* ^*tg*/*wt*^ *Csf1r* ^+/−^ mice. (B) CD19^+^ CD5^+^ CLL cell infiltration in the spleen and bone marrow in 8‐ to 10‐month‐old mice. (C) Representative immunohistochemical staining of CD45R^+^ cells in spleen sections (scale bars = 100 μm) with quantification for individual 8‐ to 10‐month‐old mice. (D) Kaplan–Meier survival curves for *TCL1* ^*tg*/*wt*^ *Csf1r* ^+/+^ and *TCL1* ^*tg*/*wt*^ *Csf1r* ^+/−^ mice. Statistical comparison was performed using the log‐rank (Mantel–Cox) test. A cohort of *n* = 8 *TCL1* ^*tg*/*wt*^ *Csf1r* ^+/+^ and *n* = 9 *TCL1* ^*tg*/*wt*^ *Csf1r* ^+/−^ mice was included for compartmental analysis in panel A and B. *n* = 5/*n* = 6 mice, respectively, were included for histology in panel D and *n* = 54/*n* = 43 mice, respectively, were included for survival analysis in panel (D).

Further analysis of lymphoid tissue infiltration between months 8 and 10 – the time point of significant reduction in peripheral blood CLL cells in *TCL1* ^*tg*/*wt*^ *Csf1r* ^+/−^ mice (Figure 3A) – showed no differences between genotypes. Spleen and liver weights were comparable between genotypes (Figure 4A), and leukaemic infiltration in spleen and bone marrow was similar (Figure 4B). Immunohistochemistry staining again confirmed comparable spleen infiltration (Figure 4C). Moreover, overall survival was also identical, with both groups living an average of 358 days (Figure 4D). Taken together, these results indicate that *Csf1r* haploinsufficiency mainly reduced CLL abundance in peripheral blood of *TCL1* ^*tg*/*wt*^ mice around the disease acceleration phase, without affecting tissue burden, and this effect diminished as leukaemia progressed.

### CSF1R‐deficient macrophages exhibit no significant alteration in their capacity to recruit and support CLL cells in vitro

**FIGURE 5:**
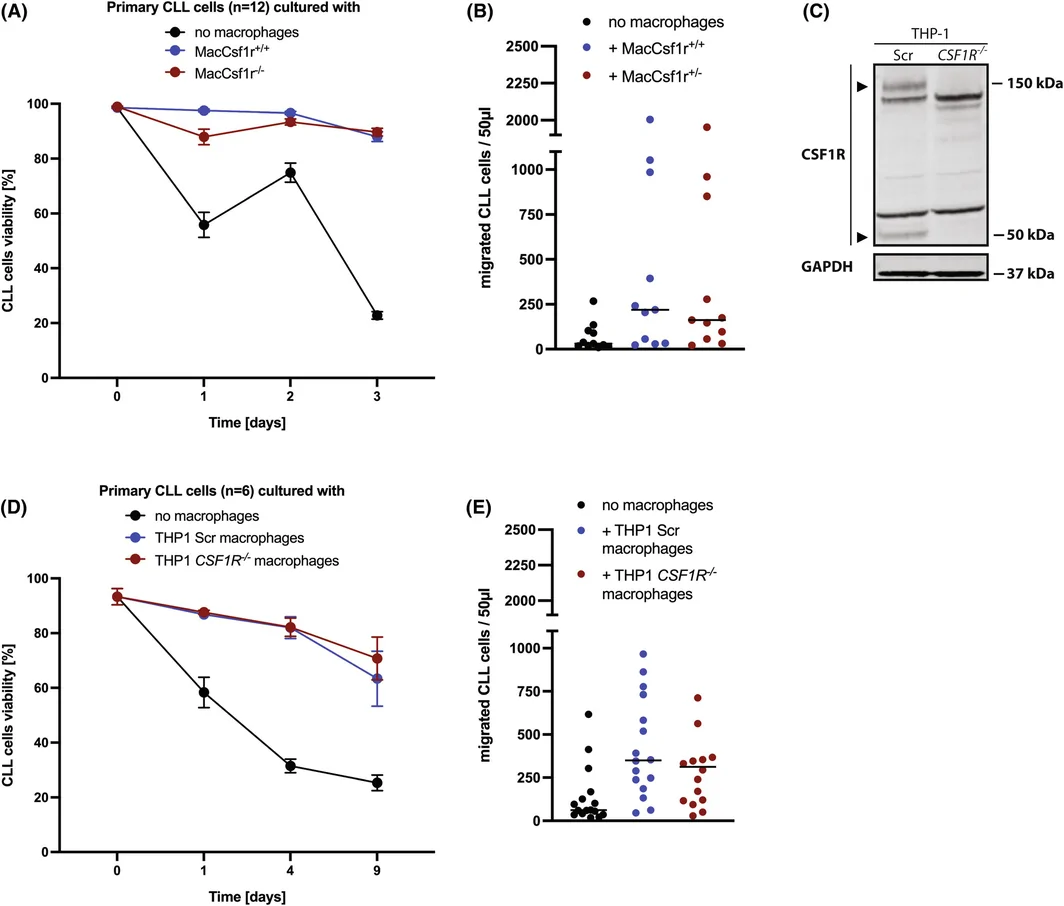
CSF1R‐deficient macrophages exhibit no significant alteration in their capacity to recruit and support CLL cells in vitro. (A) Survival of patient‐derived CLL cells co‐cultured with MacCsf1r^+/+^, MacCsf1r^−/−^ macrophages or without macrophages. Survival was assessed by Annexin V and 7‐aminoactinomycin D (7‐AAD) staining; Friedman test followed by Dunn's multiple comparisons test. (B) Migration of primary CLL cells towards MacCsf1r^+/+^, MacCsf1r^−/−^ macrophages or no macrophages, measured by flow cytometry. Data represent the mean of technical triplicates. (C) Validation of *CSF1R* knockout in THP‐1 *CSF1R* ^−/−^ cells by immunoblot analysis. The ~150 kDa and ~ 50 kDa bands correspond to mature glycosylated CSF1R and its cytoplasmic fragment respectively. The remaining bands are non‐specific. (D) Survival of patient‐derived CLL cells co‐cultured with THP‐1 Scr, (THP‐1) *CSF1R* ^−/−^ macrophages or without macrophages. Survival was assessed by Annexin V and 7‐AAD staining; Kruskal–Wallis test followed by Dunn's multiple comparisons test. (E) Migration of primary CLL cells towards THP‐1 Scr, THP‐1 *CSF1R* ^−/−^ macrophages or no macrophages, assessed as described in (B). Primary CLL cells from *n* = 12 patients for panel A, *n* = 11 patients in (B), *n* = 6 patients in (D) and *n* = 16 patients in (E) were used for in vitro assays.

To further explore the role of Csf1r in macrophage‐mediated CLL support, we co‐cultured patient‐derived CLL cells with MacCsf1r macrophages, an immortalized murine cell line lacking Csf1r expression.^29^ As we recently reported,^14^ CLL cells cultured without feeder macrophages rapidly underwent apoptosis, whereas both MacCsf1r^+/+^ and MacCsf1r^−/−^ macrophages equally supported CLL cells (Figure 5A).

Similarly, CLL migration towards MacCsf1r macrophages was unaffected by Csf1r depletion (Figure 5B). These results could be confirmed in a fully human co‐culture system of CLL cells and THP‐1 feeder macrophages.^11^, ^14^ We generated CSF1R‐deficient THP‐1 macrophages, using a CRISPR‐Cas9 mediated knockout system (Figure 5C; Figure S2). Upon co‐culture with primary human CLL, we observed neither impairment in the ability of CSF1R‐knockout macrophages to support CLL survival (Figure 5D), nor in CLL cell recruitment in vitro (Figure 5E).

**FIGURE 6:**
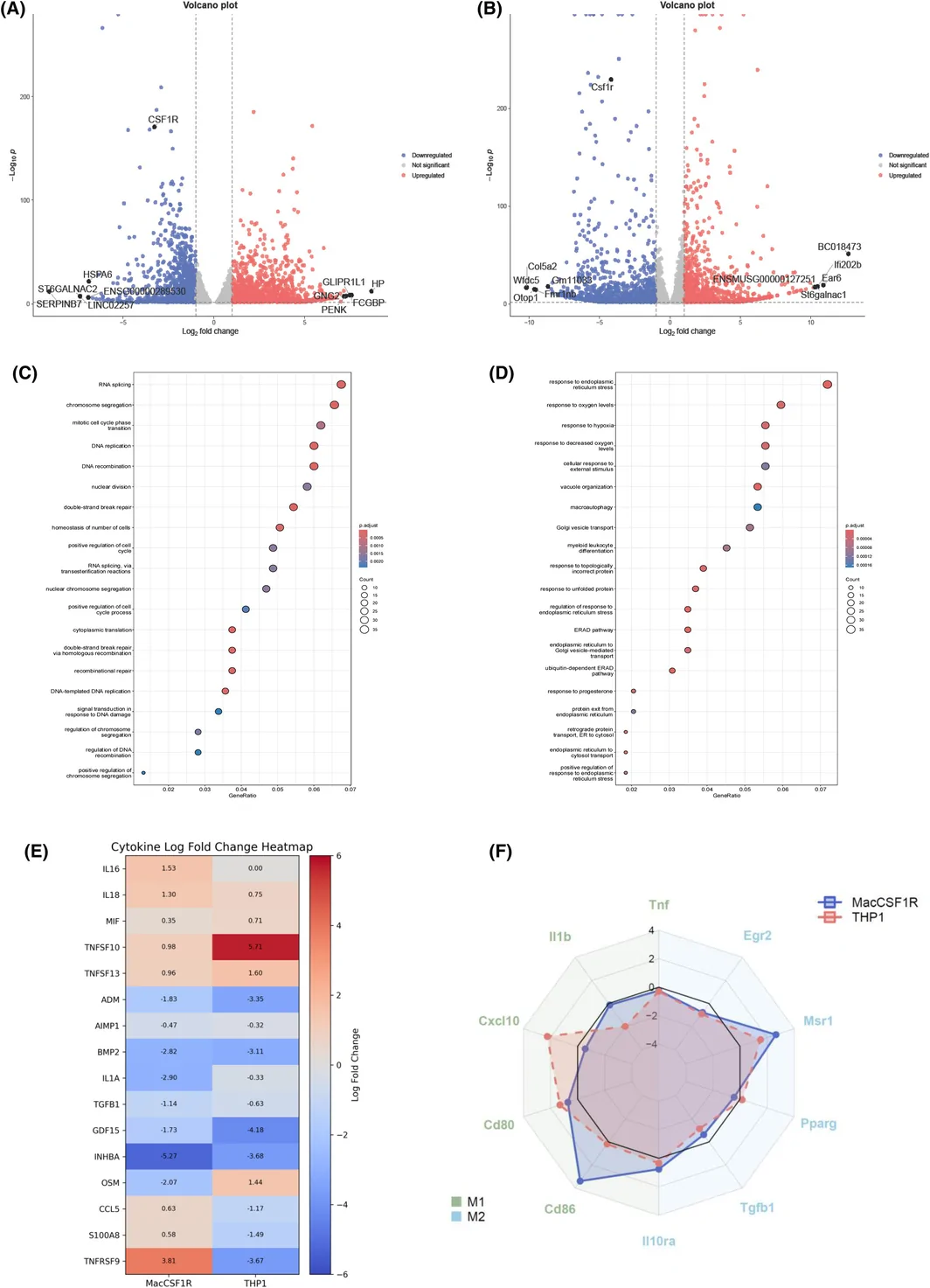
Transcriptomic profiling of CSF1R‐deficient macrophage models. (A) Volcano plot showing differentially expressed genes (DEGs) between THP‐1 Scr and THP‐1 *CSF1R* ^−/−^ macrophages. Five most highly upregulated and downregulated genes, as well as CSF1R, are highlighted. (B) Volcano plot showing DEGs between MacCsf1r^+/+^ and MacCsf1r^−/−^ cells. Five most highly upregulated and downregulated genes, as well as Csf1r (C) Gene Ontology (GO) enrichment analysis of DEGs with positive log fold change in both cell models, indicating genes more highly expressed in CSF1R knockout cells relative to controls. (D) GO enrichment analysis of DEGs with negative log fold change in both models, indicating genes more highly expressed in control cells relative to CSF1R knockout cells. (E) Heatmap of differentially expressed cytokine‐associated genes across both cell models. (F) Radar plot summarizing changes in M1‐ and M2‐associated polarization marker expression in THP‐1*CSF1R* ^−/−^ and MacCsf1r^−/−^ cells relative to corresponding controls.

To further investigate the impact of CSF1R loss on macrophage biology, we performed transcriptomic profiling of CSF1R‐deficient THP‐1 and MacCsf1r macrophages, which revealed broad transcriptional alterations in both cell models upon CSF1R loss (Figure 6A,B; Figure S3A,B). Gene Ontology (GO) and Gene Set Enrichment Analyses (GSEA) of each cell line demonstrated diverse pathways affected by CSF1R loss, reflecting their species‐specific origins (Figure S3C–F). We therefore focused on intersected differentially expressed genes (DEGs) shared between both cell models. GO analysis demonstrated enhanced signatures associated with cell proliferation, including RNA splicing, cell cycle regulation, deoxynucleic acid (DNA) replication and DNA damage repair in CSF1R‐deficient cells (Figure 6C), suggesting that these knockout cells may have acquired compensatory proliferative programmes during chronic absence of the receptor. At the same time, pathways involving responses to stimuli such as endoplasmic reticulum (ER) stress, oxygen levels, hypoxia and external stimuli were significantly downregulated in CSF1R‐deficient macrophages (Figure 6D). In addition, some pro‐inflammatory cytokines such as IL16, IL18 and MIF were increased across both CSF1R‐deficient macrophage models, whereas several members of the transforming growth factor beta (TGFβ) superfamily (TGFB1, bone morphogenetic protein 2 [BMP2], Growth differentiation factor 15 [GDF15]) were downregulated (Figure 6E), indicating a more inflammatory transcriptional profile in knockout cells. Consistently, analysis of intersected M1/M2‐associated polarization markers demonstrated enrichment of M1 markers upon CSF1R loss (Figure 6F).

Together, these in vitro results indicate that although CSF1R loss induces some increase in inflammatory and proliferative transcriptional alterations in macrophages, these changes do not impair macrophage‐mediated CLL support or recruitment, which is consistent with the unchanged CLL burden in mouse lymphoid tissues (Figure 4B,C), highlighting that the efficacy of CSF1R blockade relies mainly on myeloid cell depletion rather than direct effects of CSF1R expression on leukaemia support.

## DISCUSSION

In this study, we show that reducing CSF1R expression on myeloid cells has a limited impact on CLL progression both in vivo and in vitro. Using *Csf1r* ^+/−^ mice, which exhibit reduced receptor expression on blood monocytes while retaining normal myeloid cell counts, we observed a transient reduction in peripheral leukaemia burden at around 8 months of age in the *TCL1* ^*tg*/*wt*^ *Csf1r* ^+/−^ model. However, this effect diminished over time and did not translate into improved survival. This suggests that *Csf1r* haploinsufficiency could only partially disrupt myeloid‐mediated support, exerting minimal influence on overall CLL progression. In line with this, the complete loss of CSF1R in macrophages did not significantly affect survival or migration of patient‐derived CLL cells in vitro. Together, these results indicate that reducing Csf1r expression level could not overcome the reprogramming of tissue macrophages to support leukaemia cells in a CLL mouse model, and the therapeutic efficacy of CSF1R‐targeted strategies in CLL may depend more on the depletion of myeloid cells than on reduced CSF1R expression levels.

A limitation of the haplosufficient model is the relatively preserved Csf1r expression observed in splenic and bone marrow myeloid populations, which limits the interpretation of the full contribution of Csf1r signalling within proliferative CLL niches in vivo. Although the reduction in leukaemic burden in *TCL1* ^*tg*/*wt*^ *Csf1r* ^+/−^ mice was most pronounced at 8 months, lower disease burden was also observed at earlier time points, suggesting generally impaired disease progression. As bone marrow infiltration increases during TCL1 disease acceleration, reduced Csf1r signalling may impair establishment of supportive leukaemic niches at this stage. Consistently, we observed a trend towards reduced leukaemic proliferation in the bone marrow despite comparable infiltration levels. Impaired macrophage‐mediated pro‐survival signalling may further contribute to delayed disease progression.

Targeting the CSF1R pathway represents a clinically advanced strategy for modulating the myeloid compartment, with several inhibitors undergoing clinical trials across various malignancies.^30^, ^31^, ^32^, ^33^, ^34^ The primary goal of these therapies is the depletion of myeloid cells, particularly tumour‐associated macrophages (TAMs), whereas the biological effects of solely reducing CSF1R expression remain poorly defined. Our results with liposomal doxorubicin‐mediated monocyte depletion showed significantly impaired PBMC‐driven CLL cell viability in vitro, confirming the critical role of myeloid support in this disease. Similarly, previous murine studies demonstrated that anti‐CSF1R antibodies effectively depleted CLL‐associated macrophages, leading to redistribution of leukaemic cells into peripheral blood and enhancing sensitivity to therapies.^24^

CSF1R inhibitor activity is notably context dependent. For instance, CSF1R blockade failed to effectively deplete TAMs in glioblastoma due to compensatory GM‐CSF and interferon gamma (IFN‐γ) signalling from glioma cells.^35^ Additionally, CSF1R inhibition selectively eliminated tissue‐resident macrophages but only partially depleted splenic or lung macrophages, highlighting tissue‐specific reliance on CSF1R signalling.^36^, ^37^

In contrast to lifelong genetic haploinsufficiency, pharmacological inhibition induces acute pathway blockade and may trigger adaptive signalling responses that differ substantially from developmental compensation mechanisms observed in the transgenic mouse models. Furthermore, most small‐molecule CSF1R inhibitors target other kinases such as Fms‐like tyrosine kinase (FLT), KIT proto‐oncogene receptor tyrosine kinase (KIT) and vascular endothelial growth factor receptor (VEGFR) due to structural homology, complicating attribution of therapeutic effects solely to CSF1R inhibition. Even antibody‐based CSF1R‐targeting approaches with greater target specificity have been associated with systemic toxicities such as hepatotoxicity linked to Kupffer cell depletion, highlighting the broader physiological role of CSF1R in the tumour microenvironment. Thus, our genetic approaches, like the heterozygous or knockout models used here, provide greater specificity for dissecting CSF1R's functional role in cancer. Nevertheless, compensatory inflammatory and survival pathways may still emerge over time in response to chronic CSF1R reduction.

The CSF‐1/CSF1R axis is essential for myeloid cell homeostasis, regulating survival, differentiation and proliferation within the monocytic lineage.^38^ In CLL, elevated CSF‐1 levels likely promote monocyte expansion and polarization towards tumour‐supportive macrophages,^23^ correlating with poor prognosis.^39^ Although receptor‐mediated clearance of CSF‐1 complicates detection,^40^ *Csf1r* haploinsufficient mice do not exhibit elevated Csf‐1 levels,^17^ suggesting unsaturated clearance capacity. Despite reduced receptor levels in blood monocytes, we did not observe significant alterations in myeloid cell populations or CLL proliferation in lymphoid tissues, indicating compensatory mechanisms likely maintain microenvironmental homeostasis.

Interestingly, while confirming that Csf1r haploinsufficiency is not dosage compensated in blood monocytes,^41^ we observed no significant differences in Csf1r expression between wild‐type and Csf1r^+/−^ myeloid cells in neither spleen nor bone marrow. Likewise, myeloid populations remained stable in lymphoid niches. These results are in concordance with previous studies from the Csf1r^ΔFIRE/ΔFIRE^ mouse model. Csf1r expression is heavily dependent on the FIRE enhancer, and its deletion leads to a loss of receptor expression, but this only affects selective myeloid populations. Rojo et al. showed that heterozygous FIRE deletion reduces Csf1r expression by ~50% on blood monocytes, but not on myeloid cells from the bone marrow and, in addition, macrophage populations in spleen remain stable even in Csf1r^ΔFIRE/ΔFIRE^ mice.^42^ This may reflect compartment‐specific regulation of Csf1r expression, as tissue‐resident macrophages differ from circulating monocytes in their dependence on local niche‐derived signals and self‐renewal programmes. In addition, local Csf‐1 availability may dynamically regulate surface Csf1r abundance within tissue‐resident macrophages by ligand‐induced receptor internalization.

Notably, the transient reduction in peripheral tumour load at 8 months suggests that during the accelerated disease phase CLL cells are more dependent on microenvironmental support, including Csf1r‐expressing myeloid cells. As the disease progresses and CLL cells become more autonomous, this dependency appears to diminish, potentially explaining the reduced effect at later stages. We did not observe significant alterations in CLL proliferation and leukaemic cell distribution in lymphoid tissue, indicating compensatory mechanisms likely maintain microenvironmental homeostasis. This interpretation may also be influenced by compartment‐specific Csf1r expression, as reported in earlier studies.^42^, ^43^

Future studies employing cell‐type‐specific *Csf1r* knockout models^44^, ^45^ could further clarify distinct roles of Csf1r signalling in blood monocytes versus tissue‐resident macrophages. In particular, inducible monocyte‐specific Cre systems may enable more precise temporal and compartment‐specific investigation of Csf1r function during CLL progression. Furthermore, the adoptive transfer model of the TCL1 leukaemic clones into control versus Csf1r‐deficient recipients may provide a disease system with reduced variability compared to the TCL1 transgenic model, although they may not fully recapitulate gradual microenvironmental remodelling during leukemogenesis and the heterogeneous clinical course observed in CLL patients. Additionally, exploring interactions between CSF1R and other critical pathways such as phosphoinositide 3‐kinase/protein kinase B (PI3K/AKT) or nuclear factor kappa B (NF‐κB) ^46^, ^47^ may elucidate mechanisms of resistance and suggest novel combinatorial approaches.

Clinically, small‐molecule CSF1R inhibitors such as pexidartinib and vimseltinib are effective and Food and Drug Administration (FDA)‐approved in tenosynovial giant cell tumour (TGCT), driven by aberrant CSF‐1 production.^33^, ^48^ Outside this context, monotherapy with edicotinib or LY3022855 has shown limited clinical benefit.^25^, ^49^ However, CSF1R inhibition causes consistent depletion of TAM in the TME, including pre‐clinical models of CLL and follicular lymphoma.^24^, ^50^, ^51^ As emerging data highlight the strongly immuno‐suppressive function of TAM on T‐cell function, CSF1/CSF1R inhibition mediated TAM depletion gains increasing interest to leverage efficiency of T‐cell immunotherapy. Pre‐clinical models demonstrated that CSF1R inhibition augments CD8^+^ T‐cell tumour infiltration and increases efficiency of immune‐checkpoint inhibition in combination regimens.^52^, ^53^, ^54^ Moreover, Stahl et al. recently demonstrated that CSF1R inhibition effectively depletes TAMs and thereby strongly enhances chimeric antigen receptor T cell (CAR‐T‐cell) efficacy in a murine diffuse large B‐cell lymphoma (DLBCL) model.^55^ To date, trials combining T‐cell immunotherapy and CSF1R inhibition in solid tumours have yielded mixed outcomes.^56^, ^57^, ^58^ However, further studies, including in haematological malignancies, are still needed. In CLL, a disease characterized by dependence on tumour‐supportive macrophages and low response to T‐cell immunotherapy resulting in resistance to checkpoint blockade, combining CSF1R inhibition with emerging immunotherapies such as CAR‐T cells or bispecific antibodies might disrupt microenvironmental support and improve therapeutic outcomes.^59^ Our findings emphasize that alternative macrophage‐depleting strategies, such as nanobody‐based agents, merit further investigation as combination partners in future therapeutic approaches for CLL.

## AUTHOR CONTRIBUTIONS

**Guillermo Rodriguez‐Real:** Writing – original draft; visualization; writing – review and editing. **Thanh‐Tung Truong:** Investigation; writing – review and editing. **Shaista Ilyas:** Writing – review and editing; methodology. **Sebastian Reinartz:** Investigation; writing – review and editing. **Sanjay Mathur:** Methodology; writing – review and editing. **Luca D. Schreurs:** Investigation; visualization; writing – review and editing. **Maximilian Koch:** Investigation; writing – review and editing. **Sumiya Iqbal:** Investigation; writing – review and editing. **Alexander F. vom Stein:** Investigation; writing – review and editing. **Viktoria Kohlhas:** Investigation; writing – review and editing. **Phuong‐Hien Nguyen:** Conceptualization; writing – original draft; writing – review and editing; supervision; project administration. **Michael Hallek:** Conceptualization; funding acquisition; writing – review and editing; supervision; resources. **Natascha Rosen:** Investigation; writing – original draft; methodology; writing – review and editing; visualization. **Nina Reinart:** Conceptualization; investigation; writing – review and editing. **Trong‐Hieu Nguyen:** Formal analysis.

## FUNDING INFORMATION

This study was supported by the Deutsche Krebshilfe (DKH, German Cancer Aid) Foundation grant #70112403 to M.H. and by the Deutsche Forschungsgemeinschaft (DFG, German Research Foundation) grant SFB1530‐455784452 to M.H. and P.‐H.N.

## CONFLICT OF INTEREST STATEMENT

This study was partially supported with research funding by Gilead Sciences to M.H., N.R. is currently employed by BeiGene Germany GmbH. S.R. is currently employed by Miltenyi Biotec B.V. & Co. KG. These employments are not related to and do not influence the results of this study. The other authors declare no conflict of interest.

## Supporting information


**Figure S1.** Validation of monocyte‐dependent support of CLL cell survival.
**Figure S2.** Original unprocessed blot images for CSF1R (left) and GAPDH (right) shown in Figure 5C.
**Figure S3.** (A) PCA plot of transcriptomic profiles from THP‐1 Scr and THP‐1 *CSF1R* ^−/−^ macrophages. (B) PCA plot of transcriptomic profiles from MacCsf1r^+/+^ and MacCsf1r^−/−^ macrophages. (C) GO enrichment analysis of DEGs in THP‐1 macrophages, indicating genes more highly expressed in THP‐1 *CSF1R* ^−/−^ relative to THP‐1 Scr macrophages (upregulated in knockout cells). (D) GO enrichment analysis of DEGs in THP‐1 macrophages, indicating genes more highly expressed in THP‐1 Scr relative to THP‐1 *CSF1R* ^−/−^ macrophages (downregulated in knockout cells). (E) GO enrichment analysis of DEGs in MacCsf1r macrophages, indicating genes more highly expressed in MacCsf1r^−/−^ relative to MacCsf1r^+/+^ macrophages (upregulated in knockout cells). (F) GO enrichment analysis of DEGs in MacCsf1r macrophages, indicating genes more highly expressed in MacCsf1r^+/+^ relative to MacCsf1r^−/−^ macrophages (downregulated in knockout cells). (G) Gene set enrichment analysis (GSEA) of DEGs in THP‐1 *CSF1R* ^−/−^ relative to THP‐1 Scr macrophages. (H) Gene set enrichment analysis (GSEA) of DEGs in MacCsf1r^−/−^ relative to MacCsf1r^+/+^ macrophages.
**Table S1.** List of fluorochrome labelled antibodies used for flow cytometry experiments.
**Table S2.** List of antibodies used for immunohistochemistry staining.
**Table S3.** List of primary and secondary antibodies used in western blot analyses.

## Data Availability

The data that support the findings of this study are available from the corresponding author upon reasonable request.

## References

[1] Hallek M . Chronic lymphocytic leukemia: 2025 update on the epidemiology, pathogenesis, diagnosis, and therapy. Am J Hematol. 2025;100(3):450–480. 39871707 10.1002/ajh.27546 PMC11803567

[2] Collins RJ , Verschuer LA , Harmon BV , Prentice RL , Pope JH , Kerr JF . Spontaneous programmed death (apoptosis) of B‐chronic lymphocytic leukaemia cells following their culture in vitro. Br J Haematol. 1989;71(3):343–350. 2930721 10.1111/j.1365-2141.1989.tb04290.x

[3] Vom Stein AF , Hallek M , Nguyen PH . Role of the tumor microenvironment in CLL pathogenesis. Semin Hematol. 2024;61(3):142–154. 38220499 10.1053/j.seminhematol.2023.12.004

[4] Jestrabek H , Kohlhas V , Hallek M , Nguyen PH . Impact of leukemia‐associated macrophages on the progression and therapy response of chronic lymphocytic leukemia. Leuk Res. 2024;143:107531. 38851084 10.1016/j.leukres.2024.107531

[5] Hanna BS , Ozturk S , Seiffert M . Beyond bystanders: myeloid cells in chronic lymphocytic leukemia. Mol Immunol. 2019;110:77–87. 29173971 10.1016/j.molimm.2017.11.014

[6] Ten Hacken E , Burger JA . Microenvironment interactions and B‐cell receptor signaling in chronic lymphocytic leukemia: implications for disease pathogenesis and treatment. Biochim Biophys Acta. 2016;1863(3):401–413. 26193078 10.1016/j.bbamcr.2015.07.009 PMC4715999

[7] Burger JA , Tsukada N , Burger M , Zvaifler NJ , Dell'Aquila M , Kipps TJ . Blood‐derived nurse‐like cells protect chronic lymphocytic leukemia B cells from spontaneous apoptosis through stromal cell‐derived factor‐1. Blood. 2000;96(8):2655–2663. 11023495

[8] Jia L , Clear A , Liu FT , Matthews J , Uddin N , McCarthy A , et al. Extracellular HMGB1 promotes differentiation of nurse‐like cells in chronic lymphocytic leukemia. Blood. 2014;123(11):1709–1719. 24464016 10.1182/blood-2013-10-529610 PMC3954052

[9] Audrito V , Serra S , Brusa D , Mazzola F , Arruga F , Vaisitti T , et al. Extracellular nicotinamide phosphoribosyltransferase (NAMPT) promotes M2 macrophage polarization in chronic lymphocytic leukemia. Blood. 2015;125(1):111–123. 25368373 10.1182/blood-2014-07-589069

[10] Seiffert M , Schulz A , Ohl S , Dohner H , Stilgenbauer S , Lichter P . Soluble CD14 is a novel monocyte‐derived survival factor for chronic lymphocytic leukemia cells, which is induced by CLL cells in vitro and present at abnormally high levels in vivo. Blood. 2010;116(20):4223–4230. 20660791 10.1182/blood-2010-05-284505

[11] Nguyen PH , Fedorchenko O , Rosen N , Koch M , Barthel R , Winarski T , et al. LYN kinase in the tumor microenvironment is essential for the progression of chronic lymphocytic leukemia. Cancer Cell. 2016;30(4):610–622. 27728807 10.1016/j.ccell.2016.09.007

[12] Nguyen PH , Niesen E , Hallek M . New roles for B cell receptor associated kinases: when the B cell is not the target. Leukemia. 2019;33(3):576–587. 30700840 10.1038/s41375-018-0366-8

[13] Reinart N , Nguyen PH , Boucas J , Rosen N , Kvasnicka HM , Heukamp L , et al. Delayed development of chronic lymphocytic leukemia in the absence of macrophage migration inhibitory factor. Blood. 2013;121(5):812–821. 23118218 10.1182/blood-2012-05-431452

[14] Kohlhas V , Jestrabek H , Rebollido‐Rios R , Truong TT , von Lom A , Zölzer R , et al. Comparative analysis of macrophage feeder systems reveals distinct behaviors and key transcriptional shifts in chronic lymphocytic leukemia cells via coculture. bioRxiv. 2025;2025.02.13.638101. 10.1002/hem3.70241 PMC12558440 41158991

[15] Janowska‐Wieczorek A , Belch AR , Jacobs A , Bowen D , Padua RA , Paietta E , et al. Increased circulating colony‐stimulating factor‐1 in patients with preleukemia, leukemia, and lymphoid malignancies. Blood. 1991;77(8):1796–1803. 2015402

[16] Stanley ER , Guilbert LJ , Tushinski RJ , Bartelmez SH . CSF‐1‐‐a mononuclear phagocyte lineage‐specific hemopoietic growth factor. J Cell Biochem. 1983;21(2):151–159. 6309875 10.1002/jcb.240210206

[17] Dai XM , Ryan GR , Hapel AJ , Dominguez MG , Russell RG , Kapp S , et al. Targeted disruption of the mouse colony‐stimulating factor 1 receptor gene results in osteopetrosis, mononuclear phagocyte deficiency, increased primitive progenitor cell frequencies, and reproductive defects. Blood. 2002;99(1):111–120. 11756160 10.1182/blood.v99.1.111

[18] Chitu V , Caescu CI , Stanley ER , Lennartsson J , Rönnstrand L , Heldin C‐H . The PDGFR receptor family. In: Wheeler DL , Yarden Y , editors. Receptor tyrosine kinases: family and subfamilies. Cham: Springer International Publishing; 2015. p. 373–538.

[19] Chitu V , Gokhan S , Nandi S , Mehler MF , Stanley ER . Emerging roles for CSF‐1 receptor and its ligands in the nervous system. Trends Neurosci. 2016;39(6):378–393. 27083478 10.1016/j.tins.2016.03.005 PMC4884457

[20] Zriwil A , Boiers C , Wittmann L , Green JC , Woll PS , Jacobsen SE , et al. Macrophage colony‐stimulating factor receptor marks and regulates a fetal myeloid‐primed B‐cell progenitor in mice. Blood. 2016;128(2):217–226. 27207794 10.1182/blood-2016-01-693887 PMC5009960

[21] Tagoh H , Ingram R , Wilson N , Salvagiotto G , Warren AJ , Clarke D , et al. The mechanism of repression of the myeloid‐specific c‐fms gene by Pax5 during B lineage restriction. EMBO J. 2006;25(5):1070–1080. 16482219 10.1038/sj.emboj.7600997 PMC1409732

[22] Edwards VD , Sweeney DT , Ho H , Eide CA , Rofelty A , Agarwal A , et al. Targeting of colony‐stimulating factor 1 receptor (CSF1R) in the CLL microenvironment yields antineoplastic activity in primary patient samples. Oncotarget. 2018;9(37):24576–24589. 29872489 10.18632/oncotarget.25191 PMC5973855

[23] Polk A , Lu Y , Wang T , Seymour E , Bailey NG , Singer JW , et al. Colony‐stimulating Factor‐1 receptor is required for nurse‐like cell survival in chronic lymphocytic leukemia. Clin Cancer Res. 2016;22(24):6118–6128. 27334834 10.1158/1078-0432.CCR-15-3099 PMC5161678

[24] Galletti G , Scielzo C , Barbaglio F , Rodriguez TV , Riba M , Lazarevic D , et al. Targeting macrophages sensitizes chronic lymphocytic leukemia to apoptosis and inhibits disease progression. Cell Rep. 2016;14(7):1748–1760. 26876171 10.1016/j.celrep.2016.01.042

[25] Autio KA , Klebanoff CA , Schaer D , Kauh JSW , Slovin SF , Adamow M , et al. Immunomodulatory activity of a Colony‐stimulating Factor‐1 receptor inhibitor in patients with advanced refractory breast or prostate cancer: a phase I study. Clin Cancer Res. 2020;26(21):5609–5620. 32847933 10.1158/1078-0432.CCR-20-0855 PMC8519606

[26] Koch M , Reinartz S , Saggau J , Knittel G , Rosen N , Fedorchenko O , et al. Meta‐analysis reveals significant sex differences in chronic lymphocytic leukemia progression in the Emicro‐TCL1 transgenic mouse model. Cancers (Basel). 2020;12(7):1980. 32698538 10.3390/cancers12071980 PMC7409315

[27] Bichi R , Shinton SA , Martin ES , Koval A , Calin GA , Cesari R , et al. Human chronic lymphocytic leukemia modeled in mouse by targeted TCL1 expression. Proc Natl Acad Sci USA. 2002;99(10):6955–6960. 12011454 10.1073/pnas.102181599 PMC124510

[28] Kohlhas V , Hallek M , Nguyen PH . Constitutive activation of Lyn kinase enhances BCR responsiveness, but not the development of CLL in Emicro‐TCL1 mice. Blood Adv. 2020;4(24):6106–6116. 33351104 10.1182/bloodadvances.2020002584 PMC7756995

[29] Yu W , Chen J , Xiong Y , Pixley FJ , Dai XM , Yeung YG , et al. CSF‐1 receptor structure/function in MacCsf1r−/− macrophages: regulation of proliferation, differentiation, and morphology. J Leukoc Biol. 2008;84(3):852–863. 18519746 10.1189/jlb.0308171 PMC2516905

[30] Lei F , Cui N , Zhou C , Chodosh J , Vavvas DG , Paschalis EI . CSF1R inhibition by a small‐molecule inhibitor is not microglia specific; affecting hematopoiesis and the function of macrophages. Proc Natl Acad Sci USA. 2020;117(38):23336–23338. 32900927 10.1073/pnas.1922788117 PMC7519218

[31] Spierenburg G , Grimison P , Chevreau C , Stacchiotti S , Piperno‐Neumann S , Le Cesne A , et al. Long‐term follow‐up of nilotinib in patients with advanced tenosynovial giant cell tumours: long‐term follow‐up of nilotinib in TGCT. Eur J Cancer. 2022;173:219–228. 35932628 10.1016/j.ejca.2022.06.028

[32] Wen J , Wang S , Guo R , Liu D . CSF1R inhibitors are emerging immunotherapeutic drugs for cancer treatment. Eur J Med Chem. 2023;245(Pt 1):114884. 36335744 10.1016/j.ejmech.2022.114884

[33] Gelderblom H , Bhadri V , Stacchiotti S , Bauer S , Wagner AJ , van de Sande M , et al. Vimseltinib versus placebo for tenosynovial giant cell tumour (MOTION): a multicentre, randomised, double‐blind, placebo‐controlled, phase 3 trial. Lancet. 2024;403(10445):2709–2719. 38843860 10.1016/S0140-6736(24)00885-7 PMC11740396

[34] Gelderblom H , Razak AA , Taylor MH , Bauer TM , Wilky B , Martin‐Broto J , et al. CSF1R inhibition in patients with advanced solid tumors or Tenosynovial Giant cell tumor: a phase I study of Vimseltinib. Clin Cancer Res. 2024;30(18):3996–4004. 38995311 10.1158/1078-0432.CCR-24-0103 PMC11393540

[35] Pyonteck SM , Akkari L , Schuhmacher AJ , Bowman RL , Sevenich L , Quail DF , et al. CSF‐1R inhibition alters macrophage polarization and blocks glioma progression. Nat Med. 2013;19(10):1264–1272. 24056773 10.1038/nm.3337 PMC3840724

[36] Bosch AJT , Keller L , Steiger L , Rohm TV , Wiedemann SJ , Low AJY , et al. CSF1R inhibition with PLX5622 affects multiple immune cell compartments and induces tissue‐specific metabolic effects in lean mice. Diabetologia. 2023;66(12):2292–2306. 37792013 10.1007/s00125-023-06007-1 PMC10627931

[37] Mohamed SH , Vanhoffelen E , Shun Fu M , Hei Lau P , Hain S , Seldeslachts L , et al. CSF1R inhibition by PLX5622 reduces pulmonary fungal infection by depleting MHCII(hi) interstitial lung macrophages. Mucosal Immunol. 2024;17(6):1256–1272. 39168451 10.1016/j.mucimm.2024.08.007

[38] Stanley ER , Chitu V . CSF‐1 receptor signaling in myeloid cells. Cold Spring Harb Perspect Biol. 2014;6(6):a021857. 24890514 10.1101/cshperspect.a021857 PMC4031967

[39] Gustafson MP , Abraham RS , Lin Y , Wu W , Gastineau DA , Zent CS , et al. Association of an increased frequency of CD14+ HLA‐DR lo/neg monocytes with decreased time to progression in chronic lymphocytic leukaemia (CLL). Br J Haematol. 2012;156(5):674–676. 22050346 10.1111/j.1365-2141.2011.08902.x PMC3433277

[40] Bartocci A , Mastrogiannis DS , Migliorati G , Stockert RJ , Wolkoff AW , Stanley ER . Macrophages specifically regulate the concentration of their own growth factor in the circulation. Proc Natl Acad Sci USA. 1987;84(17):6179–6183. 2819867 10.1073/pnas.84.17.6179 PMC299033

[41] Hume DA , Caruso M , Ferrari‐Cestari M , Summers KM , Pridans C , Irvine KM . Phenotypic impacts of CSF1R deficiencies in humans and model organisms. J Leukoc Biol. 2020;107(2):205–219. 31330095 10.1002/JLB.MR0519-143R

[42] Rojo R , Raper A , Ozdemir DD , Lefevre L , Grabert K , Wollscheid‐Lengeling E , et al. Deletion of a Csf1r enhancer selectively impacts CSF1R expression and development of tissue macrophage populations. Nat Commun. 2019;10(1):3215. 31324781 10.1038/s41467-019-11053-8 PMC6642117

[43] Byrne PV , Guilbert LJ , Stanley ER . Distribution of cells bearing receptors for a colony‐stimulating factor (CSF‐1) in murine tissues. J Cell Biol. 1981;91(3 Pt 1):848–853. 6276411 10.1083/jcb.91.3.848 PMC2112824

[44] Schreiber HA , Loschko J , Karssemeijer RA , Escolano A , Meredith MM , Mucida D , et al. Intestinal monocytes and macrophages are required for T cell polarization in response to Citrobacter rodentium. J Exp Med. 2013;210(10):2025–2039. 24043764 10.1084/jem.20130903 PMC3782042

[45] Theurich S , Tsaousidou E , Hanssen R , Lempradl AM , Mauer J , Timper K , et al. IL‐6/Stat3‐dependent induction of a distinct, obesity‐associated NK cell subpopulation deteriorates energy and glucose homeostasis. Cell Metab. 2017;26(1):171–184.e6. 28683285 10.1016/j.cmet.2017.05.018

[46] Rascio F , Spadaccino F , Rocchetti MT , Castellano G , Stallone G , Netti GS , et al. The pathogenic role of PI3K/AKT pathway in cancer onset and drug resistance: An Updated Review. Cancers (Basel). 2021;13(16):3949. 34439105 10.3390/cancers13163949 PMC8394096

[47] Guo Q , Jin Y , Chen X , Ye X , Shen X , Lin M , et al. NF‐kappaB in biology and targeted therapy: new insights and translational implications. Signal Transduct Target Ther. 2024;9(1):53. 38433280 10.1038/s41392-024-01757-9 PMC10910037

[48] Tap WD , Gelderblom H , Palmerini E , Desai J , Bauer S , Blay JY , et al. Pexidartinib versus placebo for advanced tenosynovial giant cell tumour (ENLIVEN): a randomised phase 3 trial. Lancet. 2019;394(10197):478–487. 31229240 10.1016/S0140-6736(19)30764-0 PMC6860022

[49] von Tresckow B , Morschhauser F , Ribrag V , Topp MS , Chien C , Seetharam S , et al. An open‐label, multicenter, phase I/II study of JNJ‐40346527, a CSF‐1R inhibitor, in patients with relapsed or refractory Hodgkin lymphoma. Clin Cancer Res. 2015;21(8):1843–1850. 25628399 10.1158/1078-0432.CCR-14-1845

[50] Gomez‐Roca CA , Italiano A , Le Tourneau C , Cassier PA , Toulmonde M , D'Angelo SP , et al. Phase I study of emactuzumab single agent or in combination with paclitaxel in patients with advanced/metastatic solid tumors reveals depletion of immunosuppressive M2‐like macrophages. Ann Oncol. 2019;30(8):1381–1392. 31114846 10.1093/annonc/mdz163 PMC8887589

[51] Valero JG , Matas‐Cespedes A , Arenas F , Rodriguez V , Carreras J , Serrat N , et al. The receptor of the colony‐stimulating factor‐1 (CSF‐1R) is a novel prognostic factor and therapeutic target in follicular lymphoma. Leukemia. 2021;35(9):2635–2649. 33731849 10.1038/s41375-021-01201-9 PMC8410584

[52] Zhu Y , Knolhoff BL , Meyer MA , Nywening TM , West BL , Luo J , et al. CSF1/CSF1R blockade reprograms tumor‐infiltrating macrophages and improves response to T‐cell checkpoint immunotherapy in pancreatic cancer models. Cancer Res. 2014;74(18):5057–5069. 25082815 10.1158/0008-5472.CAN-13-3723 PMC4182950

[53] Peranzoni E , Lemoine J , Vimeux L , Feuillet V , Barrin S , Kantari‐Mimoun C , et al. Macrophages impede CD8 T cells from reaching tumor cells and limit the efficacy of anti‐PD‐1 treatment. Proc Natl Acad Sci USA. 2018;115(17):E4041–E4050. 29632196 10.1073/pnas.1720948115 PMC5924916

[54] Sato T , Sugiyama D , Koseki J , Kojima Y , Hattori S , Sone K , et al. Sustained inhibition of CSF1R signaling augments antitumor immunity through inhibiting tumor‐associated macrophages. JCI Insight. 2025;10(1):e178146. 39782686 10.1172/jci.insight.178146 PMC11721313

[55] Stahl D , Godel P , Balke‐Want H , Gholamipoorfard R , Segbers P , Tetenborg L , et al. CSF1R(+) myeloid‐monocytic cells drive CAR‐T cell resistance in aggressive B cell lymphoma. Cancer Cell. 2025;43(8):1476–1494.e10. 40513575 10.1016/j.ccell.2025.05.013

[56] Weiss SA , Djureinovic D , Jessel S , Krykbaeva I , Zhang L , Jilaveanu L , et al. A phase I study of APX005M and Cabiralizumab with or without Nivolumab in patients with melanoma, kidney cancer, or non‐small cell lung cancer resistant to anti‐PD‐1/PD‐L1. Clin Cancer Res. 2021;27(17):4757–4767. 34140403 10.1158/1078-0432.CCR-21-0903 PMC9236708

[57] Razak AR , Cleary JM , Moreno V , Boyer M , Calvo Aller E , Edenfield W , et al. Safety and efficacy of AMG 820, an anti‐colony‐stimulating factor 1 receptor antibody, in combination with pembrolizumab in adults with advanced solid tumors. J Immunother Cancer. 2020;8(2):e001006. 33046621 10.1136/jitc-2020-001006 PMC7552843

[58] Foster CC , Fleming GF , Karrison TG , Liao CY , Desai AV , Moroney JW , et al. Phase I study of stereotactic body radiotherapy plus Nivolumab and Urelumab or Cabiralizumab in advanced solid tumors. Clin Cancer Res. 2021;27(20):5510–5518. 34168049 10.1158/1078-0432.CCR-21-0810

[59] Lewis RI , Vom Stein AF , Hallek M . Targeting the tumor microenvironment for treating double‐refractory chronic lymphocytic leukemia. Blood. 2024;144(6):601–614. 38776510 10.1182/blood.2023022861

