# Supplementary material for: Reduced colony‐stimulating factor 1 receptor expression in myeloid cells has limited impact on chronic lymphocytic leukaemia progression
Source: Br J Haematol. 2026 Jul 26;209(3):939–52. doi: 10.1111/bjh.70700 (PMC13570146; doi:10.1111/bjh.70700)
Supplement: Supplementary file 1 [file BJH-209-939-s001.docx]

**Reduced CSF1R expression in myeloid cells has limited impact on chronic lymphocytic leukemia progression**

Natascha Rosen^1^, Guillermo Rodriguez-Real^1^, Alexander F. vom Stein^1^, Luca D. Schreurs^1^, Trong-Hieu Nguyen^1,2^, Viktoria Kohlhas^1^, Thanh-Tung Truong^1^, Maximilian Koch^1^, Sebastian Reinartz^1^, Sumiya Iqbal^3^, Shaista Ilyas^3^, Sanjay Mathur^3^, Nina Reinart^1^, Phuong-Hien Nguyen^1,^*, and Michael Hallek^1,^*

^1^ University of Cologne, Faculty of Medicine and University Hospital Cologne, Department I of Internal Medicine, Center for Integrated Oncology Aachen Bonn Cologne Duesseldorf; Center for Molecular Medicine Cologne; CECAD Center of Excellence on Cellular Stress Responses in Aging-Associated Diseases, 50931 Cologne, Germany

^2^ Institute of Molecular Medicine, RWTH Aachen University, Aachen, Germany

^3^ Institute of Inorganic and Materials Chemistry, University of Cologne, Greinstr. 6, 50939 Cologne, Germany; Center for Molecular Medicine Cologne

**Supplemental Methods**

**Hematologic Analysis**

Complete blood counts were obtained using a Sysmex XE-5000 hematology analyzer (Sysmex Corporation), which quantifies white blood cells (WBCs) via impedance and optical scatter without antibody labeling. For flow cytometry, red blood cells (RBCs) were lysed with ACK buffer, and the remaining leukocytes were washed and resuspended in PBS.

**Flow cytometry**

Single-cell suspensions were stained with fluorochrome-conjugated antibodies (Supplementary Table 1) as previously described (11, 13, 28). Data were acquired using a MACSQuant VYB cytometer (Miltenyi Biotec) and analyzed with Kaluza (Beckman Coulter) or FlowJo (BD Pharmingen). Mean fluorescence intensity (MFI) was calculated as indicated in the respective figure legends.

**Immunohistochemistry**

Spleen tissues from 8- to 10-month-old mice were fixed in 4% buffered paraformaldehyde and paraffin-embedded. Sections were stained with antibodies listed in Supplementary Table 2, following the manufacturers’ instructions. Signal detection was performed using the Histofine® Simple Stain Mouse MAX PO polymer-based system (Nichirei Biosciences).

**Cell proliferation assay**

Peripheral blood, bone marrow and spleen were collected from 8- to 10-month-old mice. To prepare single cell suspensions from bone marrow, both tibia and femur were flushed with PBS. Spleen cells were passed through a 100-μm cell strainer (Greiner Bio-One) and RBCs were lysed using ACK lysing buffer, washed and resuspended in PBS. Intracellular Ki-67 staining was performed using IntraPrep Permeabilization Reagent (Beckman Coulter) following manufacturer’s instructions. For specific antibodies, see Supplementary Table 1.

**Western blot**

Cells were lysed in RIPA buffer with 1 mM PMSF (Cell Signaling), incubated on ice for 1 hour, centrifuged, and supernatants stored at −80 °C. Immunoblots were performed per manufacturer’s instructions using NuPAGE 4–12% Bis-Tris gels (Thermo Scientific) and semi-dry transfer to nitrocellulose membranes (GE Healthcare). Signals were detected by immunofluorescence on a LiCor Odyssey CXl. Antibodies are listed in Supplementary Table 3.

**CLL migration assays towards macrophages**

MacCsf1r cells (1 × 10⁵) were seeded in 24-well plates. After 24 h, medium was replaced with RPMI + 10% FBS, 1% Pen/Strep, and 110 ng/mL GM-CSF. After 20 h, serum-starved CLL cells (1 × 10⁶, 2 h) were added to transwell inserts (5 μm pores, Corning). After 4 h, migrated cells were counted by flow cytometry (MACSQuant X, Miltenyi Biotec) and analyzed with FlowJo (BD Pharmingen).

For THP-1 cells, 1 × 10⁵ cells were differentiated into macrophages by stimulation with 100 ng/mL PMA for 48 hours. After differentiation, THP-1 macrophages were washed twice with PBS and 1 × 10⁶ CLL cells were added, and the same protocol previously described for MacCsf1r cells was followed.

**References**

**Supplemental Figures**

**Supplementary Figure 1. Validation of monocyte-dependent support of CLL cell survival.**

(A) Survival of total peripheral blood mononuclear cells (PBMCs) from healthy donors and distinct immune cell subsets within these PBMCs (CD14⁺ monocytes, CD4⁺ T cells, CD8⁺ T cells, and CD20⁺ B cells), after in vitro treatment for 24 hours with DMSO (control) or liposomal doxorubicin (Lipo-Doxo) at 0.1 µM or 1 µM. Cell viability was assessed by Annexin V staining and flow cytometry, and values were normalized to the respective DMSO control for each population. Statistical analysis was performed using the Wilcoxon test.

(B) Survival of patient-derived CLL cells co-cultured with healthy donor PBMCs that were pre-treated in vitro for 24 hours with DMSO or 1 µM Lipo-Doxo. Co-cultures were maintained for 24, 48 and 72 hours, after which the survival of CLL cells (CD5⁺ CD19⁺) was assessed as in (A). Non-normalized data were used for analysis.

**Supplementary Figure 2.** Original unprocessed blot images for CSF1R (left) and GAPDH (right) shown in Figure 5C.

**Supplementary Figure 3.**

(A) PCA plot of transcriptomic profiles from THP-1 Scr and THP-1 *CSF1R^-/-^* macrophages.

(B) PCA plot of transcriptomic profiles from MacCsf1r^+/+^ and MacCsf1r^-/-^ macrophages.

(C) GO enrichment analysis of DEGs in THP-1 macrophages, indicating genes more highly expressed in THP-1 *CSF1R^-/-^* relative to THP-1 Scr macrophages (upregulated in knockout cells).

(D) GO enrichment analysis of DEGs in THP-1 macrophages, indicating genes more highly expressed in THP-1 Scr relative to THP-1 *CSF1R^-/-^* macrophages (downregulated in knockout cells).

(E) GO enrichment analysis of DEGs in MacCsf1r macrophages, indicating genes more highly expressed in MacCsf1r^-/-^ relative to MacCsf1r^+/+^ macrophages (upregulated in knockout cells).

(F) GO enrichment analysis of DEGs in MacCsf1r macrophages, indicating genes more highly expressed in MacCsf1r^+/+^ relative to MacCsf1r^-/-^ macrophages (downregulated in knockout cells).

(G) Gene set enrichment analysis (GSEA) of DEGs in THP-1 *CSF1R^-/-^* relative to THP-1 Scr macrophages.

(H) Gene set enrichment analysis (GSEA) of DEGs in MacCsf1r^-/-^ relative to MacCsf1r^+/+^ macrophages.

**Supplemental Tables**

**Table S1: List of fluorochrome labelled antibodies used for flow cytometry experiments.**

| **Antibody** | **Manufacturer** | **Reference** |
| --- | --- | --- |
| CD5 | Miltenyi Biotec | Clone 53-7.3 |
| CD19 | Miltenyi Biotec | Clone 6D5 |
| CD45 | Miltenyi Biotec | Clone 30F11 |
| CD115 | Miltenyi Biotec | Clone AFS98 |
| CD115 Isotype control antibody (rat IgG2a) | Miltenyi Biotec | Clone ES26-15B7.3 |
| CD11b | BioLegend | Clone M1/70 |
| F4/80 | BioLegend | Clone BM8 |
| Ki-67 | BioLegend | Clone 16A8 |
| Ki-67 Isotype control antibody (Rat IgG2a) | BioLegend | Clone RTK2758 |

**Table S2: List of antibodies used for immunohistochemistry staining.**

| **Antibody** | **Manufacturer** | **Reference** |
| --- | --- | --- |
| CD68 | Abcam | ab125212 |
| CD45R | BD Bioscience | Clone RA3-6B2 |

**Table S3: List of primary and secondary antibodies used in Western Blot analyses.**

| **Antibody** | **Manufacturer** | **Reference** |
| --- | --- | --- |
| CD115 (CSF1R) | Cell Signaling | #3152 |
| GAPDH (D16H11) | Cell Signaling | #5174 |
| IRDye® 680LT anti-rabbit IgG Donkey | LI-COR | #926-68023 |
